# Clinical Utility of Early Intervention Including the 5-Step Precision Medicine Method in First-Episode Psychosis: Protocol for a Cohort Study With Nested Economic and Process Evaluations

**DOI:** 10.2196/74408

**Published:** 2025-09-23

**Authors:** Jesús Pérez, David Heredero Jung, Óscar Gonzalo, Belén García Berrocal, Carmen García Cerdán, Pablo Salas Aranda, Alejandro de la Sota Pérez, Sandra Milagros Lorenzo Hernández, Elena Marcos Vadillo, Rocío García García, Vanesa Berdión Marcos, Ana Maciá Casas, Belén Refoyo Matellán, Berta Bote Bonaechea, Llanyra García Ullán, Carolina Lorenzo Romo, Carmen Martín Gómez, Concha Turrión Gómez, María Isidoro García

**Affiliations:** 1 Universidad de Salamanca Salamanca Spain; 2 Instituto de Investigación Biomédica de Salamanca (IBSAL) Salamanca Spain; 3 Hospital Universitario de Salamanca Salamanca Spain

**Keywords:** first-episode psychosis, early intervention, precision medicine, personalized precision psychiatry, antipsychotics, schizophrenia

## Abstract

**Background:**

Psychotic disorders such as schizophrenia present a significant challenge to health care systems due to their high disability rates and treatment costs. With discontinuation rates for antipsychotics reaching over 40% in the first year and 80% after 3 years, it is crucial to tailor antipsychotic selection and dosing early in treatment. Personalized precision psychiatry, underpinned by pharmacogenetics, holds considerable potential in individualizing antipsychotic treatment for patients with first-episode psychosis. An internationally pioneering method called 5-step precision medicine (5SPM) focuses on the application of pharmacogenetics to clinical practice. The recently launched Prevention and Early Intervention in Mental Health (PRINT) program in Salamanca, Spain, integrates this method to enhance early intervention for adolescents and young people with first-episode psychosis.

**Objective:**

The Clinical Utility of Early Intervention Including the 5SPM Method in First-Episode Psychosis (CLUMP) project aims to explore whether an early intervention model of personalized precision psychiatry including pharmacogenetics improves adherence to antipsychotic medicines and, therefore, clinical and functional outcomes in young people experiencing the first episode of a psychotic illness.

**Methods:**

To achieve our objectives, we shall compare adherence to the first prescribed antipsychotic medication and clinical and functional outcomes between patients with first-episode psychosis. We shall compare 2 cohorts: cohort 1 will receive the recently introduced PRINT program including the 5SPM method, and cohort 2 will have received standard care provided by mental health services before the PRINT program implementation. The primary outcome to measure treatment adherence will be all-cause discontinuation proportions during the 1-year follow-up. Secondary outcome measures will include pragmatic efficacy, tolerability, and functional outcome measures. For additional comparative purposes, we shall analyze the environmental, clinical, and pharmacogenetic information of patients with psychotic disorders of more than 5 years of evolution and with other mental disorders whose data are currently stored and have been ethically approved for research use. A total of 300 patients will be included in the study. Analyses will include descriptive statistics, comparison tests, Kaplan-Meier survival curves, multivariate log rank tests, qualitative analysis, and cost-benefit evaluation.

**Results:**

Ethics approval was obtained in June 2023. Recruitment for the CLUMP project began in January 2025, and enrollment for cohort 1 will continue until May 2026. All data collection is expected to be completed by June 2027. Data analyses are estimated to take approximately 6 months. The project is scheduled to conclude in December 2027.

**Conclusions:**

The CLUMP project is set to provide the first clear blueprint for implementing and evaluating the impact of personalized precision psychiatry based on pharmacogenetics in the context of early intervention programs for the benefit of young people experiencing the first episode of a severe mental illness such as schizophrenia.

**International Registered Report Identifier (IRRID):**

DERR1-10.2196/74408

## Introduction

### Background

Psychotic illnesses such as schizophrenia and related conditions are a major issue for public health care systems; they cause severe disability and are expensive to treat. As the illness becomes manifest during adolescence or young adulthood, nonspecific signs such as anxiety, depression, and emerging psychotic features form a prelude to the full psychotic syndrome that may last for months or even years before patients seek help or receive treatment. This duration of untreated psychosis is inversely related to outcome. Therefore, providing effective treatments as soon as possible, shortening the duration of untreated psychosis, may prevent the development of a more severe illness, improve quality of life, and lead to recovery in many cases [1,2]. Many people at an early stage of their psychotic illness, in addition to delusions or hallucinations, may develop cognitive problems that underpin disability. These impairments contribute to problems in functional outcomes that are considered by patients and their relatives to be as important as the phenomena that professionals focus on. Furthermore, the evolution of psychotic disorders varies hugely and raises the question of why certain people improve and others stay unwell despite having gone through similar interventions [1].

The development of specific psychological and pharmacological treatments to tackle first presentations of psychotic illnesses has been relatively slow. However, services to deliver existing evidence-based interventions to people at that early clinical stage have changed dramatically over the last 2 decades. Traditional multidisciplinary community mental health teams now work as specialized early intervention in psychosis teams. Seminal work, especially from Australia and the United Kingdom, has led to widespread adoption of these assertive treatment programs for young people in the initial phases of psychotic illnesses. In this context, rapid access and adherence to effective pharmacological treatments, specifically antipsychotics, play a fundamental role in “the shorter the DUP, the better the outcome” motto that early intervention in psychosis is predicated upon [1,2]. In fact, there is a plethora of evidence demonstrating that adherence to antipsychotic treatment has a positive impact on psychotic illness progression and longer-term outcomes such as hospital admissions, functioning, and suicide rates [3-5].

The introduction of early intervention programs has led clinicians toward a more accurate identification of the psychological and social interventions that each patient can benefit from; however, clinicians still face difficulties in selecting the antipsychotic medicine that better suits their patients [6-8]. Thus far, pragmatic randomized controlled trials have been the main source of scientific information to decide on the most effective and tolerable antipsychotics for patients with first-episode psychosis. These studies have found all-cause discontinuation rates for individual antipsychotics of approximately 40% or above during the first year of follow-up, which increases to 80% after 3 years of treatment even in the context of traditional early intervention in psychosis services [7,8]. These results fall short in terms of shifting clinicians’ trial-and-error approach to prescribing antipsychotics in clinical practice, which often ends in changes from those that do not yield sufficient therapeutic response or cause adverse effects [4]. This is further complicated by the fact that patients’ early experiences of antipsychotics nurture long-term perceptions of them; therefore, experiencing side effects or a lack of efficacy can make young individuals develop negative attitudes toward these drugs [9]. In fact, the high prevalence of adverse reactions in young, drug-naïve patients and lack of therapeutic response from antipsychotics are significant contributors to treatment discontinuation [9,10]. This stresses the need to tailor antipsychotic (and dose) selection to each patient from the very early stages of their psychotic illness.

While the use of pharmacogenetics (the effect of genetic factors on reactions to medicines) in people treated with antipsychotics has already found strong associations between treatment responses, side effects, and genetic variations, studies describing the clinical utility of pharmacogenetics are still very scarce and mainly limited to patients with severe and enduring psychotic disorders and poor response to treatment [11-13]. Notably, the systematic implementation of pharmacogenetics and its clinical effect on those experiencing the first episode of a psychotic illness has still not been tested. Personalized precision psychiatry using pharmacogenetics could help individualize antipsychotic treatment for patients with first-episode psychosis. It could reduce the time needed to find a tolerable and effective antipsychotic drug for people that were never exposed to such medications. Pharmacogenetics would simply be another tool that early intervention services could adopt and implement within their treatment pathways to improve medication adherence [13].

At the Salamanca University Healthcare Complex (CAUSA) in Spain, specifically in the Salamanca University Hospital (HUS), our unit of pharmacogenetics and precision medicine has been working on the application of pharmacogenetics to clinical practice for more than a decade [14]. We have developed an internationally pioneering method called 5-step precision medicine (5SPM) that addresses most of the implementation challenges outlined previously. The award-winning 5SPM model is based on updated scientific knowledge, integrating new technologies from up-to-date, highly efficient genotyping to state-of-the-art bioinformatics and artificial intelligence [13-17]. In fact, the 5SPM model is paving the way for the implementation of pharmacogenetics across different clinical specialties. In contrast to other existing methodologies, which are mostly guided by laboratory genetic results, the 5SPM method also addresses the specific needs and characteristics of the patient whom it is applied to, taking into consideration clinical and epidemiological variables as well as any other factor, such as polypharmacy, that could affect treatment response. Operationally, it is structured in the following five steps: (1) collection of clinical, epidemiological, and treatment data, including current prescriptions, diagnosis, and treatment response; (2) study of pharmacological interactions based on drug-specific pharmacokinetic pathways by processing databases such as the Pharmacogenomics Knowledge Base, PubMed–National Center for Biotechnology Information, Charite’s SuperCYP-Transformer, and the Pharmacogene Variation Consortium; (3) pharmacogenetic analysis of the genes that code enzymes involved in drug-specific metabolism; (4) modification or introduction of pharmacotherapy according to the data obtained in the previous 3 steps; and (5) analysis of the treatment outputs and, depending on clinical evolution, reassessment if needed.

The 5SPM model relies on a multidisciplinary approach, implying that technical and clinical staff must work closely together, and strictly adheres to ethical requirements such as comprehensive informed consent. This method has already shown quantifiable positive impact on the quality of life of patients in different clinical fields, including psychiatry. Since its inception, more than 1000 patients have benefited from it, and it has also generated additional phamacogenetics knowledge [13,16,18]. This is corroborated by a significant scientific body of evidence supporting the effectiveness and cost-efficiency of this method in real-world settings when compared to treatment as usual. The 5SPM approach has been successfully implemented in diverse psychiatric populations, including individuals with chronic psychotic disorders, bipolar disorder, and complex multimorbid presentations involving polypharmacy [13-19]. Clinical protocols guided by the 5SPM approach integrate pharmacogenetic data—particularly cytochrome P450 (CYP450) genotyping—with a patient’s clinical, pharmacological, and biological history to optimize treatment selection. In patients with poor response to antipsychotics, this approach has identified high rates of gene-drug and drug-drug interactions, allowing for targeted therapeutic adjustments that significantly reduced polytherapy and daily dosages, thereby lowering the risk of adverse effects and enhancing adherence [13]. Moreover, in broader psychiatric cohorts, pre-emptive pharmacogenetic testing has shown utility in preventing affective switching in bipolar disorder and mitigating side effects associated with selective serotonin reuptake inhibitor treatment based on cytochrome P2D6 and P2C19 metabolizer status [18]. These interventions have not only improved clinical outcomes but also demonstrated economic value, with cost-benefit ratios exceeding 3:1 in some studies and consistent reductions in hospitalization and treatment costs across patient groups [19]. Collectively, these findings support the clinical scalability of the 5SPM approach and its potential to enhance safety, efficacy, and cost-efficiency in precision psychiatry.

### Objectives

We have fully integrated the 5SPM method into our recently launched Prevention and Early Intervention in Mental Health (PRINT) program and shall evaluate its impact. The PRINT program is a novel clinical service that aims to provide early, comprehensive care to adolescents and young adults experiencing first-episode psychosis in Salamanca. This is one of only a few services of this kind in Spain, where early intervention is still not widespread. Considering that there is still a marked heterogeneity in treatment engagement and response among patients with first-episode psychosis in early intervention services worldwide [20], the PRINT program is refreshing the early intervention model by optimizing pharmacological choices for such a clinical group following the 5SPM methodology. Thus, the Clinical Utility of Early Intervention Including the 5SPM Method in First-Episode Psychosis (CLUMP) project aims to explore whether an early intervention model of personalized precision psychiatry including pharmacogenetics improves adherence to antipsychotic medicines and, therefore, clinical and functional outcomes in young people experiencing the first episode of a psychotic illness.

We hypothesize that integrating the 5SPM method into PRINT will significantly improve adherence to antipsychotic treatment and, consequently, enhance both clinical and functional outcomes.

The CLUMP project’s objectives will be to (1) introduce an innovative early intervention model of personalized precision psychiatry including the 5SPM method for patients with first-episode psychosis; (2) ascertain whether such a model can reduce the elevated discontinuation rates of antipsychotic medications among this group within the first year of follow-up by clinical services; (3) assess the impact of the new model on pragmatic efficacy measures such as hospital admissions and functional outcomes such as return to work or education; (4) determine whether this innovation can bring cost benefits to the public health care system and the wider economy; and (5) establish a blueprint for implementing this precision medicine model, specifically pharmacogenetics, across early intervention in psychosis programs nationally and internationally.

## Methods

### Study Design

To test our hypothesis and achieve our objectives, we shall compare adherence to the first prescribed antipsychotic medication and pragmatic clinical and functional outcomes between 2 cohorts of patients with first-episode psychosis. One cohort will comprise patients treated before the implementation of an early intervention in psychosis model of personalized precision psychiatry, including pharmacogenetics, and the other will comprise patients treated under this new model. In addition, we shall compare the pharmacogenetic profiles and possible phenocopies (variations of phenotypes produced environmentally, eg, due to polypharmacy, rather than genetically) between these patients with first-episode psychosis and a national sample of patients with longer-term psychotic disorders or other mental health conditions to evaluate potential implications for clinical management at different stages of a psychotic illness and across mental disorders.

### Participants

A total of 300 patients will be included in this project. We shall include 2 different cohorts. Cohort 1 will be a prospective sample of patients with first-episode psychosis referred over 18 months to the new early intervention in psychosis program of personalized precision psychiatry (PRINT) at CAUSA. Cohort 2 will be a retrospective, consecutive (in reverse chronological order as registered in the CAUSA electronic health records) sample of patients who experienced a first episode of psychosis before the implementation of PRINT.

Inclusion and exclusion criteria for both cohorts will be aligned with the PRINT program referral acceptance criteria, which are predominantly inclusive.

Inclusion criteria are as follows: patients (1) with a diagnosis of first-episode psychosis, either nonaffective or affective; (2) aged 12 to 35 years; and (3) followed by clinical services for at least 1 year or earlier if there was evidence of antipsychotic treatment discontinuation (see the Outcome Measures section). In addition, patients in cohort 1 or their family or legal representatives will have to provide written consent to take part in the CLUMP project, including pharmacogenetics analysis (see the Ethical Considerations section). For patients in retrospective cohort 2, this will not be an inclusion criterion, but all of them will be approached by our research team seeking their consent to obtain their pharmacogenetic profile for research purposes.

Exclusion criteria are as follows: patients (1) with first-episode psychosis due to organic causes, for example, brain diseases such as Huntington or Parkinson disease, HIV, syphilis, dementia, brain tumors or cysts, or brain injury; (2) with moderate to severe intellectual disability; and (3) who spent (cohort 2) or plan to reside during (cohort 1) most of the 1-year follow-up period in a locality out of the PRINT program’s catchment area.

For additional comparative purposes, we shall analyze the environmental, clinical, and pharmacogenetic information of patients with psychotic disorders of more than 5 years of evolution or with other mental disorders whose data are currently stored and have been ethically approved for research use in our unit of pharmacogenetics and precision medicine at the HUS.

### Clinical Management and Pharmacogenetics Analysis

Patients in cohort 1 will receive the recently introduced PRINT program, an early intervention in psychosis model, including the 5SPM method based on pharmacogenetics developed by our unit of pharmacogenetics and precision medicine at the HUS. As mentioned previously, the 5SPM method includes five steps: (1) collection of clinical, epidemiological, and treatment data; (2) study of pharmacological interactions based on drug-specific pharmacokinetic pathways; (3) pharmacogenetic analysis of the genes that code enzymes involved in drug-specific metabolism; (4) introduction of or change in pharmacotherapy according to the data obtained in the previous 3 steps; and (5) analysis of the treatment outputs and reassessment if needed. These steps will be preceded by an informed consent process. This preliminary procedure plus steps 1 to 4 will be conducted as soon as possible, ideally when a patient with a first episode of psychosis is identified by clinical services, aiming to minimize the time between the initial presentation and pharmacogenetics-informed decisions on pharmacotherapy. Step 5 (treatment review) will be carried out 6 months after antipsychotic treatment initiation. All steps will require a multidisciplinary approach and a shared decision-making process including patients or their family or legal representatives, especially if the patient lacks the mental capacity to make such decisions.

For the pharmacogenetics analysis (step 3), we shall use mass spectrometry and real-time polymerase chain reaction to study the genes encoding enzymes 1A2, 2B6, 2C9, 2C19, 2D6, 3A4, and 3A5 of the CYP450 family, in addition to transporter ABCB1. The CYP450 superfamily contains highly polymorphic genes. The usual approach to the relationship between genotypic variability and the metabolism of CYP450 substrates is based on the definition of metabolic phenotypes with their characteristic pharmacokinetic implications according to different genetic mechanisms. Metabolic profiles will be considered to make decisions on antipsychotic treatment choices. We already have enough technical support and pharmacogenetics evidence for most antipsychotics used in the early stages of a psychotic illness (ie, olanzapine, risperidone, paliperidone, quetiapine, aripiprazole, amisulpride, lurasidone, cariprazine, haloperidol, and zuclopenthixol).

Cohort 2 will have received standard care provided by mental health services before the PRINT program implementation, specifically by mental health professionals working in generic community mental health teams that offer assessment and treatment to people who reside in their geographical catchment areas and experience a wide range of mental disorders, not only psychotic disorders.

### Patient and Public Involvement

Patients have been involved in the design and will participate in the delivery of this trial. For instance, patients with first-episode psychosis were consulted on the feasibility of running this project, and some of them were involved in its design, specifically on what information to include in the informed consent form to carry out the pharmacogenetics analyses. In addition, we shall constitute a CLUMP patient and carer advisory group, which will also contribute to the identification of themes for the qualitative evaluation (see the Qualitative Evaluation section).

### Outcome Measures

#### Primary Outcome Measure

The primary outcome to measure treatment adherence will be all-cause discontinuation of the initially prescribed antipsychotic medication following the patient’s or treating clinician’s decision. All-cause discontinuation rates in the CLUMP project will be defined by all-cause discontinuation proportions during the 1-year follow-up and the mean time to such discontinuation.

For cohort 1, the 1-year follow-up period will start when an antipsychotic medication is prescribed taking into consideration the pharmacogenetics advice emerging from the 5SPM method. For cohort 2, follow-up would begin on the date when the first antipsychotic medication was prescribed. For both cohorts, the first prescribed antipsychotic will be the initially prescribed antipsychotic drug for maintenance treatment, not only for short-term use to control psychomotor agitation. In addition, in accordance with existing scientific consensus, the discontinuation date will be defined as the first day of a 2-week or longer interruption of the first prescribed antipsychotic. In cohort 2, treatment discontinuation dates will be determined by examining clinical records. Where only approximate dates are available, the first, middle, or final day of the month will be used as best estimates. In cases in which cross-titration of medication took place, the date of discontinuation will be the first day of the cross-tapering process.

All-cause discontinuation will also be stratified by 4 reasons: insufficient efficacy, adverse events, lack of adherence, and other. Insufficient efficacy will be established at the treating clinician’s judgment only after at least 3 weeks of treatment. If more than one reason for discontinuation was present, we shall select one by applying the ranking order as stated previously.

#### Secondary Outcome Measures

We shall also collect pragmatic efficacy, tolerability, and functional outcome measures in both cohorts. These will include evaluations during the follow-up period regarding hospital admissions; visits to accident and emergency departments and outpatient clinics; changes in and number of medications; changes in antipsychotic dose over time; use of chlorpromazine dose equivalents as reference; type and number of side effects, including impact on BMI; and initiation or return to work or academic activities.

As part of routine practice, patients in cohort 1 will also be assessed using the quality-of-life self-completion questionnaire EQ-5D-5L to determine overall improvement over time. Scores from this questionnaire will also feed into the 5SPM step 5 to make treatment decisions at month 6. This cohort will also be evaluated using a battery of questionnaires, including the Positive and Negative Syndrome Scale [21], Calgary Depression Scale for Schizophrenia [22], Brief Negative Symptom Scale [23], and Udvalg for Kliniske Undersøgelser Side Effect Rating Scale [24].

### Effect and Sample Size

We conducted a power calculation for the main outcome: the proportion of patients with first-episode psychosis who, by their treating clinician’s or their own decision, discontinue, for any reason, the first prescribed antipsychotic medication within 1 year. As stated previously, recent research studies, mostly carried out in early intervention settings, concur that 40% of patients with first-episode psychosis experience first prescribed antipsychotic drug discontinuation during the first year following treatment initiation [7,8]. This proportion may be even higher in patients receiving mainstream psychiatric care, such as our cohort 2. Considering the expectations behind the use of personalized precision medicine, PRINT should aim to reduce discontinuation rates to 10%. Therefore, for a power of 80% with (1) a significance level of .05 (2-sided), (2) all-cause discontinuation rate in cohort 1 (intervention) of 0.4, (3) all-cause discontinuation rate in cohort 2 (treatment as usual) of 0.1, and (4) an expected dropout or loss to follow-up rate of 0.2, our calculations would require a sample size of at least 38 patients in each cohort. We used the R statistical software *pwr* package for this calculation (version 4.4.2 for Windows; R Foundation for Statistical Computing).

In CAUSA, we have found an administrative incidence of 50 new patients aged 12 to 35 years every year. Thus, we would expect at least 75 patients eligible to be recruited for cohort 1 over 18 months. Using a conservative approach, if 25 patients (approximately 35%) do not agree to take part, we would still enroll 50 patients. This would be in addition to another 50 patients in our retrospective cohort 2 and pharmacogenetic data currently available at CAUSA from 100 patients with psychotic disorders of more than 5 years of evolution and another 100 patients with other mental disorders that we shall use for further comparative purposes, as outlined previously. This all accounts for a total of 300 participants contributing to this project.

### Statistical Analysis

Comparisons of the main outcome (ie, discontinuation rates) will be carried out using the chi-square test. Kaplan-Meier survival curves and multivariate log rank tests will be used to assess time to all-cause antipsychotic discontinuation. For these analyses, patients will be followed up on for a year, from antipsychotic treatment initiation until discontinuation. Thus, we shall censor survival time by the end of the 1-year observation period or before such date if there is enough evidence of antipsychotic discontinuation. We shall describe and compare sociodemographic information and clinical and functional outcomes within and between groups. Means and SDs will be used to describe quantitative variables. We shall use the chi-square test or Fisher exact test for categorical variables and Mann-Whitney *U* test for continuous variables, among others. Data analyses will be conducted using the R statistical software (version 4.4.2 for Windows).

### Qualitative Evaluation

We shall aim to interview 15 patients from cohort 1 to obtain their views on the PRINT program, specifically on the implementation of the 5SPM method. We shall recruit participants who complete the 1-year follow-up but also some who might withdraw from treatment before then. In principle, the interviews will explore the following thematic questions: (1) To what extent the goals of personalized precision psychiatry based on pharmacogenetics were aligned with the patients’ own goals? (2) Did the PRINT program help in achieving their goals, what did not, and why? (3) Is there anything that could have been performed differently during the informed consent and 5SPM processes? (4) What might have contributed to nonengagement and treatment discontinuation? (5) What would help them meet their goals and priorities better and why?

As stated in the Patient and Public Involvement section, the CLUMP patient and carer advisory group will also contribute to the identification of additional themes for the qualitative evaluation.

Sampling will be purposive, with quota stratification by gender and clinical background, to enhance transferability through thick description of participant characteristics and context. The CLUMP project team will also be interviewed to characterize their perspectives on implementing the new model and triangulate experiences of model implementation. An independent researcher trained in qualitative methods will conduct all the interviews, which will be audio recorded and transcribed verbatim. Data analysis will be based on the constant comparative method [25]. This will involve intensive engagement with the data using comparison across datasets to generate higher-order themes with explanatory value. An audit trail—including coding logs, analytical memos, and interview minutes—will be maintained to support dependability and confirmability of findings. To strengthen credibility, this study will use data triangulation (patients and professionals), peer debriefing within the research team, and member checking with the patient and carer advisory group [26,27]. The NVivo software (QSR International) will be used to assist in coding the data.

### Economic Evaluation

We shall explore cost-benefit using the primary outcome measure: all-cause discontinuation. The time horizon will be 1 year after antipsychotic initiation for both cohorts. Costs and outcomes will be compared at the final follow-up point and presented as mean values with SDs by cohort. The costs will be estimated using service use data. We shall collect these data using an adapted Spanish-language version of the Early Intervention Adult Service Use Schedule [28], which contains costs that are easy to track and calculate in the Spanish health system. The Early Intervention Adult Service Use Schedule will be completed for both cohorts. This measure covers the use of all health and social care services over 12 months (with an interim collection at 6 months), as well as sociodemographic information and employment status. In cohort 1, we shall also conduct a cost-utility analysis using quality-adjusted life years derived from the EQ-5D-5L.

### Data Management

Data collected for the CLUMP project will be narrative and numeric and will be obtained from interviews, review of clinical records, and blood tests. They will include sociodemographic information; pragmatic outcome measures; questionnaires to measure efficacy, functioning, and tolerability; and pharmacogenetic information.

As with all clinical data, the findings of this study will be kept confidential to protect personally identifiable information. To ensure that all information collected about participants remains confidential, we will only use anonymized participant information. Codes linking the identity of the participants to clinical data will be stored on a secure password-protected database. Personal data needed to contact the participants will be securely stored on a separate database. Only the project team and professionals working at the clinical services that the individuals are recruited from or at the unit of precision medicine and pharmacogenetics at the HUS will have access to the participants’ personal data. Only nonidentifiable participant data will be transferred to a central database for the project, and only nonidentifiable information will be made available to any other researchers or collaborators involved in the study. All data will be encrypted and stored in secure databases on a secure network using established procedures. All study personnel will have to be certified to conduct research involving human participants and will be aware of the importance of maintaining strict confidentiality. Clinical notes will not be accessed by those outside the direct health care team, which includes members of the research team, until consent from participants is in place. Any hard copies of all data and records, including consent forms, will be stored in locked filing cabinets in locked rooms in security-protected buildings. Electronic and hard copies containing data from the project will be stored at CAUSA buildings and electronic facilities for a minimum of 10 years in accordance with good research practice. The storage of blood sample leftovers will be addressed in accordance with the informed consent form signed by the patients and current legislation.

The final responsibility over the use of personal data will rest with the Specialized Clinical Assistance Managerial Office at CAUSA, and the data protection officer at the Regional Ministry of Health will be contactable by all personnel involved and research participants. All data processing will be subject to the Spanish Organic Law 3/2018 for personal data and digital rights protection and the European Union General Data Protection Regulation 2016/679. General Data Protection Regulation articles 6 (on lawfulness of data processing) and 9 (on processing of special categories of personal data, such as genetic information) will be particularly relevant for this project. In addition, participants will have the right to have access to and rectify their data or, eventually, limit or oppose their use under the terms included in articles 15 to 23. They can execute these rights before the principal investigator for this project and the data protection officer. Participants will be informed that they can also present complaints with regard to the use of their data to the Spanish Agency of Data Protection.

### Ethical Considerations

Approval for this project was obtained from the Ethics Committee for Research With Medicines of the Salamanca Health Area (reference PI 2023 06 1308) on June 24^th^ 2023. Written informed consent will be obtained from all participants and their legal representatives to ensure that they understand the study requirements, risks, and benefits. If potential participants accept an invitation to participate in the study, they will be provided with a participant information sheet and consent form for consideration and sign off. There will not be economic compensation for participating in the study.

## Results

**Figure 1:**
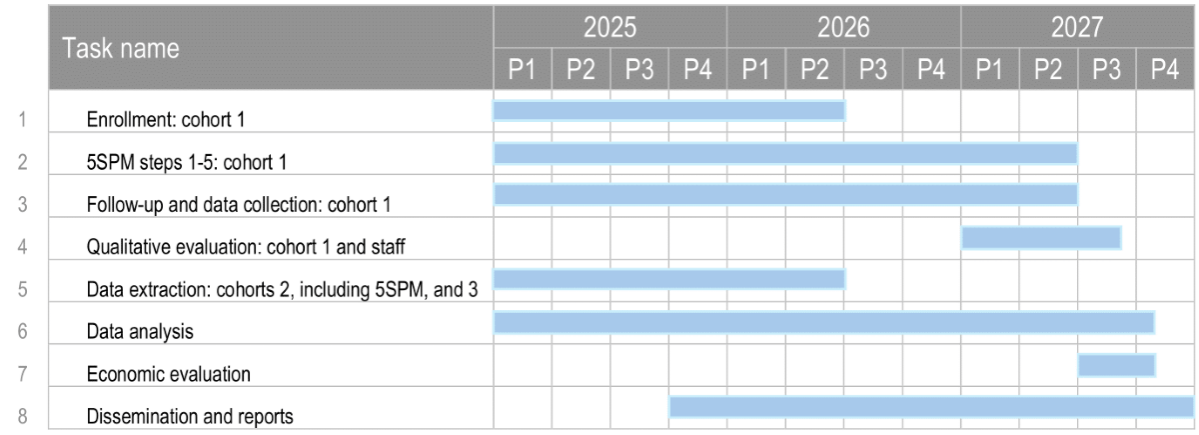
The Clinical Utility of Early Intervention Including the 5-Step Precision Medicine (5SPM) Method in First-Episode Psychosis (CLUMP) project Gantt chart.

Recruitment for the CLUMP project began in January 2025, and enrollment for cohort 1 will continue until May 2026. All data collection is expected to be completed by June 2027. Data analyses are estimated to take approximately 6 months. The project is scheduled to conclude in December 2027. Figure 1 provides a schematic Gantt chart for the project, including timelines for each task.

## Discussion

### Expected Findings

The CLUMP project aims to improve the adherence and outcomes of patients with first-episode psychosis through the implementation and evaluation of an innovative early intervention program of personalized precision psychiatry based on pharmacogenetics.

To date, the uptake of pharmacogenetics testing in psychiatry has been limited to very few contributions and mainly focused on affective disorders such as depression [6,11]. This is at odds with strategies worldwide planning to implement precision medicine as part of policies to enhance the uptake of genomic-driven medicine, including in psychiatry. For instance, the UK National Health Service has set up the Genomic Medicine Service with the aim of making pharmacogenetics part of routine clinical practice. Recently, the Royal Dutch Association for the Advancement of Pharmacy, Dutch Pharmacogenetics Working Group, and Clinical Pharmacogenetics Implementation Consortium have developed evidence-based pharmacogenetics prescribing guidelines, which are compiled in the Pharmacogenomics Knowledge Base and rated by gene-drug pairing level of evidence, ie, how confident we are that a person's genetic variation affects their response to a specific drug [6,11,29,30]. These guidelines include antipsychotics and recommend that clinicians consider genotyping of patients who experience inefficacy or side effects from any of the drugs that have enough pharmacogenetic evidence. This information could then be used to personalize prescribing decisions. Pharmacogenetics in psychiatry has mainly focused so far on pharmacokinetics, and there is consensus that genetic information related to enzymes of the CYP450 family, such as cytochromes P2D6 and P2C19, can be used to provide treatment advice. In fact, it is estimated that between 36% and 62% of the population have variants of these 2 isoenzymes that may modify the metabolism of psychiatric medications [6,31]. However, although current pharmacogenetics guidelines are used by an increasing number of mental health professionals, they do not take polypharmacy into consideration, and their full integration into mental health care is still a worldwide challenge [6].

Even though patients and clinicians perceive the value of pharmacogenetics in mental health and the potential positive impact on patients’ engagement with and trust in both the prescribed medication and the prescriber, certain barriers preclude its successful implementation. For instance, mental health professionals feel that they lack pharmacogenetics theoretical and practical knowledge (ie, how to make use of it as part of their practice) and have concerns about how to integrate it within their workloads without increasing consultation times or delaying treatment decisions for too long. They worry about this genetic information replacing clinical judgment or the risk of misinterpretations by mixing up the strength of drug-gene interactions with overall drug effectiveness and not paying enough attention to other important environmental and biological factors that play a role in treatment response. In addition, this innovation is not free of ethical considerations, such as the potential psychological consequences of disclosing pharmacogenetics results to patients or matters related to genetic privacy. Furthermore, research studies have rarely measured the clinical effects or cost-benefit associated with pharmacogenetics application, and existing guidelines mostly advise on the pharmacogenetics of individual drugs without considering the common use of polypharmacy in clinical practice [6,11]. This all reflects, to a large extent, the lack of clear methodologies to embed and measure the impact of pharmacogenetics in day-to-day psychiatric practice.

Therefore, the problem that we strive to solve is cultural, linked to prevailing trial-and-error prescribing of antipsychotics in clinical practice, as well as structural, concerning the evidence-policy-practice gap for tailoring drug treatments to patients with psychotic disorders at the earliest opportunity. Nonetheless, our new joint venture of early intervention in psychosis and applied precision medicine, combined with our previous experience using the 5SPM method for patients with mental disorders, should give us scientific and logistic traction to achieve our aims. Indeed, a guided implementation of pharmacogenetics for real-world clinical settings following the 5SPM methodology could help overcome patients’ and clinicians’ uncertainties and facilitate the use of this evidence-based innovation not only to address poor response to or adverse events from psychiatric medications in patients with established psychotic disorders but also to promote personalized and pre-emptive drug prescription [11]. The CLUMP project should be able to provide the first clear blueprint for implementing and evaluating the impact of personalized precision psychiatry based on pharmacogenetics in the context of early intervention programs for the benefit of young people experiencing the first episode of a severe mental illness such as schizophrenia.

### Limitations

As we will carry out the CLUMP project in 1 center, this might affect the generalizability of our results. Nevertheless, Salamanca has a university that attracts more than 40,000 students within our participants’ age range every year and is a province with a diverse young population, some of them living in socially deprived areas. These characteristics will temper this limitation. On the other hand, given our project’s originality, we considered it important to test our hypothesis in a relatively circumscribed location and, later, move toward its implementation at scale. In addition, our prospective cohort 1 may be affected by a status quo bias (ie, attempts to maintain pharmacogenetically guided prescription despite lack of efficacy or presence of adverse events). This will be controlled through other pragmatic outcome measures and the 5SPM step 5 at month 6, which will include questionnaires such as the EQ-5D-5L to inform treatment maintenance or change.

## Appendix

Multimedia Appendix 1 Peer review report from Carlos III Health Institute (Instituto de Salud Carlos III), General Subdirectorate of Evaluation and Research Promotion, Ministry of Science and Education, Spain.

## Abbreviations

5SPM: 5-step precision medicine
CAUSA: Salamanca University Healthcare Complex
CLUMP: Clinical Utility of Early Intervention Including the 5-Step Precision Medicine Method in First-Episode Psychosis
CYP450: cytochrome P450
HUS: Salamanca University Hospital
PRINT: Prevention and Early Intervention in Mental Health
