# Supplementary material for: Clinical Utility of Early Intervention Including the 5-Step Precision Medicine Method in First-Episode Psychosis: Protocol for a Cohort Study With Nested Economic and Process Evaluations
Source: JMIR Res Protoc. 2025 Sep 23;14:e74408. doi: 10.2196/74408 (PMC12504900; doi:10.2196/74408)
Supplement: Multimedia Appendix 1 [file resprot_v14i1e74408_app1.pdf]

Validación CSV

Copia válida para su uso en papel. El documento original ha sido firmado por:
SELLO DEL INSTITUTO DE SALUD CARLOS III Fecha: 20/10/2023 15:38:45
CSV del documento original: ISCIII6578936085456863561 URL: https://firmadoc.isciii.es/CSV/ISCIII6578936085456863561

DATOS GENERALES

**EXPEDIENTE**	PI23/00582
**INVESTIGADOR PRINCIPAL**	PEREZ SANCHEZ-TOLEDO, JESUS
**COINVESTIGADOR PRINCIPAL**	ISIDORO GARCIA, MARIA
**TÍTULO**	Utilidad clínica de la intervención temprana combinada con el método de medicina de precisión de 5 pasos (5SPM) en el primer episodio psicótico: El Proyecto CLUMP
**CENTRO DE REALIZACIÓN**	INSTITUTO DE INVESTIGACION BIOMEDICA DE SALAMANCA (IBSAL)
**CENTRO SOLICITANTE**	INSTITUTO DE INVESTIGACION BIOMEDICA DE SALAMANCA (IBSAL) DE LA FIECSCYL
**COMISIÓN TECNICA DE EVALUACIÓN**	Enfermedades neurológicas y mentales

INFORME CIENTÍFICO-TÉCNICO (máx. 50 puntos)

A) VALORACIÓN DEL EQUIPO DE INVESTIGACIÓN:(máx. 20 puntos en este apartado):

1º Perfil del IP /COIP (De 0-12 puntos): dar hasta 8 puntos para proyectos que cumplan con los criterios y subir a 12 sólo en casos de recorridos excelentes de los IP/COIP.

2º Equipo investigador: (De 0 a 5 puntos): adecuación entre competencias/capacidades en el ámbito de conocimiento del proyecto y las actividades previstas para su desarrollo.

3º Capacidad formativa del IP/COIP y del equipo: (De 0 a 3 puntos): tener en cuenta para valorar este ítem: dirección de Tesis Doctorales, dirección de Máster; captación de financiación de RR. HH. en convocatorias competitivas, si tiene residentes o rotantes a su cargo.

Justifique brevemente la valoración otorgada en cada uno de los tres apartados, respecto a la **valoración científica del equipo**

El IP presenta un excelente recorrido internacional, con publicaciones en revistas de renombre. Ha obtenido financiación competitiva de diversas fuentes como IP. La co-IP tiene una loable trayectoria científica en el campo de la medicina personalizada y de precisión. Ha participado en numerosos artículos en revistas de alto impacto. No se evidencia una colaboración previa, y no participan en estructuras estables de investigación.
Tanto IP como co-IP aportarían conocimiento preciso y altamente especializado, por lo que sus perfiles son claramente complementarios y aditivos. El equipo propuesto aporta conocimientos específicos que benefician la propuesta.
Tanto IP como Co-IP tienen un claro perfil formativo, con dirección activa de tesis doctorales y acompañamiento de profesionales en formación. El IP declara haber obtenido becas de investigación internacionales que incluían recursos humanos (RRHH) en categorías similares a las consideradas por el Instituto de Salud Carlos III.

Validación CSV

Copia válida para su uso en papel. El documento original ha sido firmado por:
SELLO DEL INSTITUTO DE SALUD CARLOS III Fecha: 20/10/2023 15:38:45
CSV del documento original: ISCIII6578936085456863561 URL: https://firmadoc.isciii.es/CSV/ISCIII6578936085456863561

B) VALORACIÓN DEL PROYECTO: (máx. 30 puntos en este apartado):

1º Calidad científica: (De 0 a 7 puntos): novedad de la propuesta, originalidad en el abordaje conceptual y/o experimental de la misma, pertinencia; efecto incentivador, análisis actualizado del estado de conocimiento; integración en una línea de investigación estable con proyectos previos que se detallan; capacidad de transferencia directa de los resultados esperados.

Justifique la valoración otorgada

La propuesta tiene una elevada calidad científica, destacando la novedad y originalidad del acercamiento 5SMP en primeros episodios de psicosis, ya que hasta el momento se había utilizado en otros trastornos mentales. Se considera que podría haber sido de interés haber especificado más hallazgos en otros trastornos mentales crónicos para contextualizar el impacto esperado. Este nuevo modelo implicaría un factor clave en la adherencia al tratamiento en una población donde esta es de crucial relevancia para la progresión y pronóstico del cuadro, denotando su gran pertinencia.

2º Calidad metodológica (De 0 a 8 puntos): alineamiento entre hipótesis y objetivos, formulados de forma clara y concisa; adecuación metodológica del diseño, variables y plan de análisis estadístico a los objetivos propuestos; tamaño muestral, justificación formal/informal; identificación de fuentes de sesgos y previsión para su control; propuesta de plan de gestión de datos (PGD).

Justifique la valoración otorgada

La presentación de los objetivos y plan de trabajo es clara y adecuada, aunque la hipótesis se considera genérica y sin estar planteada en relación a cada uno de los objetivos. El tamaño muestral está calculado y justificado en función de la discontinuación esperada. Las variables de medida están numeradas, sin embargo, podría clarificarse cómo se obtendrán las mismas especialmente aquellas que forman parte de los objetivos del proyecto como por ejemplo como se va a medir el funcionamiento de esta población. Además, podría ser de interés añadir una escala específica para medir la adherencia.
Se destaca positivamente la propuesta de incorporar al estudio un grupo de individuos con experiencia en primeros episodios de psicosis, ya sea pacientes o cuidadores: The CLUMP Patient and Carer Advisory Group, y que el mismo tenga una perspectiva de género.

Validación CSV

Copia válida para su uso en papel. El documento original ha sido firmado por:
SELLO DEL INSTITUTO DE SALUD CARLOS III Fecha: 20/10/2023 15:38:45
CSV del documento original: ISCIII6578936085456863561 URL: https://firmadoc.isciii.es/CSV/ISCIII6578936085456863561

3º Viabilidad: (De 0 a 15 puntos): adecuación del equipo al proyecto, adecuación entre sus competencias y las actividades experimentales a desarrollar, experiencia previa; plan de trabajo, distribución de tareas y cronograma; identificación de riesgos y planificación de su control; infraestructuras disponibles, pertinencia y capacidad para su gestión; adecuación del presupuesto solicitado a los objetivos del proyecto; experiencia acreditada en gestión de proyectos, valoración específica en propuestas coordinadas o con presupuesto solicitado>300.000 euros.

Justifique la valoración otorgada

El proyecto es factible de ser llevado a cabo, se describen correctamente los paquetes de trabajo y las actividades en las que estarán implicados los participantes del proyecto. Las actividades que cada uno realizará están acordes a las áreas de conocimiento. Se considera excesivo solicitar dos contratos teniendo en cuenta los miembros del equipo presentados en el proyecto, y el elevado número de doctorandos que podrían participar en el proyecto. El presupuesto para las publicaciones se considera desmesurado.

Justifique brevemente la respuesta **respecto a valoración científica del proyecto**. Señale el grado de adecuación entre relevancia, aplicabilidad y capacidad de transferencia.

Se trata de un proyecto relevante e innovador que busca reducir las elevadas tasas de discontinuación del tratamiento antipsicótico prescrito. El proyecto está diseñado dentro del marco de la medicina de precisión y el desarrollo y la puesta en práctica de nuevas tecnologías diagnósticas y terapéuticas.

VALORACIÓN FINAL

El proyecto ha sido valorado positivamente, ya que demuestra alta calidad científica. Está liderado por dos profesionales referentes en sus respectivas áreas de conocimiento, enriqueciendo los potenciales resultados. Los objetivos son de una inconmensurable relevancia clínica, debido al periodo de la enfermedad en el que se centrarán los investigadores, momento donde la adherencia farmacológica es crucial para el pronóstico de los pacientes. De obtener resultados positivos, se estaría cada vez más cerca de la aplicabilidad de la medicina de precisión en la práctica clínica de la psiquiatría. El presupuesto podría ajustarse tanto en personal como en publicaciones.

(Los datos personales que aparecen en este documento sólo serán utilizados para los fines previstos en la correspondiente Convocatoria o para su tratamiento automatizado con fines estadísticos con las garantías previstas en Reglamento (UE) 2016/679 del Parlamento Europeo y del Consejo de 27, de abril de 2016, relativo a la protección de las personas físicas en lo que respecta al tratamiento de datos personales y a la libre circulación de estos datos y por el que se deroga la Directiva 95/46/CE (Reglamento general de protección de datos) y en la Ley Orgánica 3/2018, de 5 de diciembre, de Protección de Datos Personales y garantía de los derechos digitales)

Fecha: jueves, 6 de julio de 2023

Validación CSV

Copia válida para su uso en papel. El documento original ha sido firmado por:
SELLO DEL INSTITUTO DE SALUD CARLOS III Fecha: 20/10/2023 15:38:49
CSV del documento original: ISCIII9769120985456860459 URL: https://firmadoc.isciii.es/CSV/ISCIII9769120985456860459

DATOS GENERALES

**EXPEDIENTE**	PI23/00582
**INVESTIGADOR PRINCIPAL**	PEREZ SANCHEZ-TOLEDO, JESUS
**COINVESTIGADOR PRINCIPAL**	ISIDORO GARCIA, MARIA
**TÍTULO**	Utilidad clínica de la intervención temprana combinada con el método de medicina de precisión de 5 pasos (5SPM) en el primer episodio psicótico: El Proyecto CLUMP
**CENTRO DE REALIZACIÓN**	INSTITUTO DE INVESTIGACION BIOMEDICA DE SALAMANCA (IBSAL)
**CENTRO SOLICITANTE**	INSTITUTO DE INVESTIGACION BIOMEDICA DE SALAMANCA (IBSAL) DE LA FIECSCYL
**COMISIÓN TÉCNICA DE EVALUACIÓN**	Enfermedades neurológicas y mentales

INFORME ESTRATÉGICO Y DE OPORTUNIDAD (máx 50 puntos)

A) Valoración del equipo de investigación (máx. 10 puntos).

Justifique brevemente la respuesta respecto a valoración estratégica del equipo de investigación.

IP y CO IP con acreditada trayectoria investigadora en cuanto a generación de proyectos competitivos e impacto bibliométrico, el IP principalmente en el Reino Unido , donde fue director del servico de intervención temprana CAMEO (Cambridge Assessing, Managing and Enhancing Outcomes), por el que tuvo el reconocimiento de Premio Nacional de Excelencia Clínica e Investigadora por el Ministerio de Sanidad Británico. LA CO-IP tha ocupado diversos cargos de de representación y coordinación de comisiones oficiales de investigación. La Unidad que dirige ha desarrollado desde hace más de 10 años el método 5SPM (5-Step Precision Medicine -Medicina de Precisión de 5 Pasos), recientemente galardonado con el premio internacional de Biomedicina Rafael Hervada

B) Valoración del proyecto (máx. 40 puntos).

1º Relevancia: (De 0 a 15 puntos): contribución esperada al avance de la ciencia en salud; estimación formal/informal del impacto (en salud, económico y social) por la implementación de sus resultados.

Justifique brevemente la respuesta.

El proyecto CLUMP está centrado en mejorar la adherencia a fármacos antipsicóticos y los resultados terapéuticos en estos pacientes. Pretende introducir un modelo pionero de intervención temprana de Psiquiatría Personalizada de Precisión mediante farmacogenética para pacientes con Primer Episodio Psicòtico (PEP) y determinar si este modelo puede reducir la elevada discontinuación de antipsicóticos en este grupo de pacientes

2º Aplicabilidad: (De 0 a 15 puntos): aplicabilidad directa o diferida, justificar la aplicabilidad directa, en caso de aplicabilidad diferida, considerar si se identifican retos regulatorios/organizativos a su implementación; vulnerabilidad del problema abordado; requiere modificaciones regulatorias u organizativas mayores.

Validación CSV

Copia válida para su uso en papel. El documento original ha sido firmado por:
SELLO DEL INSTITUTO DE SALUD CARLOS III Fecha: 20/10/2023 15:38:49
CSV del documento original: ISCIII9769120985456860459 URL: https://firmadoc.isciii.es/CSV/ISCIII9769120985456860459

Justifique brevemente la respuesta.

El proyecto tiene una elevada aplicabilidad pues quiere comprobar si la introducción del programa de Psiquiatría Personalizada de Precisión para primeros episodios psicóticos, que incluye el método 5SPM, puede reducir drásticamente las tasas de discontinuación del tratamiento antipsicótico prescrito inicialmente.

3º Capacidad de transferencia del proyecto: (De 0 a 8 puntos): actuaciones previstas orientadas a la transferencia a la práctica clínica de los resultados, y su potencial incorporación en cartera de servicios; transferencia al sector productivo; limitaciones mayores para la transferencia efectiva.

Justifique brevemente la respuesta.

Aunque la farmacogenética busca personalizar los tratamientos, las respuestas individuales pueden seguir siendo variables, lo que limita la certeza de resultados uniformes. La implementación de la medicina de precisión y la farmacogenética avanzada puede requerir recursos significativos en términos de equipamiento, capacitación y personal especializado, lo que podría limitar su transferibilidad en entornos con recursos limitados.

4º Perspectiva de género: (De 0 a 2 puntos): ¿está incorporada en el diseño? ¿Es adecuado el planteamiento?

Justifique brevemente la respuesta.

Equidad de género en el equipo y mención al estudio diferencial por género.

Justifique brevemente la respuesta respecto a la valoración estratégica del proyecto. Señale el grado de adecuación entre relevancia, aplicabilidad y capacidad de transferencia.

Equipo con IP y coIP con proyecto ambicioso llevado a cabo previamente en Reino Unido. A pesar de su alta valoración estratégica en la intersección de relevancia, aplicabilidad y capacidad de transferencia, el proyecto enfrenta posibles limitaciones en términos de la complejidad de su metodología combinada, el desafío de coordinar profesionales multidisciplinarios que no han trabajado en equipo previamente.

Validación CSV

Copia válida para su uso en papel. El documento original ha sido firmado por:
SELLO DEL INSTITUTO DE SALUD CARLOS III Fecha: 20/10/2023 15:38:49
CSV del documento original: ISCIII976912098545686O459 URL: https://firmadoc.isciii.es/CSV/ISCIII976912098545686O459

VALORACIÓN

Grupo con solvencia acreditada que une miembros de neurociencia clínica que estudia prevención, detección temprana e intervención en salud mental, liderado por el Prof. Jesús Pérez (IP), y miembros del área de genética molecular y medicina de precisión, liderada por la Dra. María Isidoro, en el grupo de inmunología y enfermedades inflamatorias (CoIP). El proyecto demuestra una alta valoración estratégica al presentar una alineación entre su relevancia en abordar el primer episodio psicótico, su aplicabilidad al combinar la intervención temprana y la farmacogenética avanzada, y su capacidad de transferencia potencial a través del enfoque de medicina de precisión. La integración de métodos innovadores como el método de 5 pasos y la farmacogenética confiere al proyecto un alto potencial para impactar positivamente la atención clínica y la personalización del tratamiento en trastornos psicóticos, destacando su robusta estrategia en la intersección de relevancia, aplicabilidad y transferibilidad. El presupuesto empleado en personal se tendria que reformular.

Fecha: martes, 22 de agosto de 2023

Los datos personales que aparecen en este documento sólo serán utilizados para los fines previstos en la correspondiente Convocatoria o para su tratamiento automatizado con fines estadísticos con las garantías previstas en Reglamento (UE) 2016/679 del Parlamento Europeo y del Consejo de 27, de abril de 2016, relativo a la protección de las personas físicas en lo que respecta al tratamiento de datos personales y a la libre circulación de estos datos y por el que se deroga la Directiva 95/46/CE (Reglamento general de protección de datos) y en la Ley Orgánica 3/2018, de 5 de diciembre, de Protección de Datos Personales y garantía de los derechos digitales.

D. Luis García Ortiz, con DNI 07833414M, Director Científico del Instituto de Investigación Biomédica de Salamanca (IBSAL),

CERTIFICA:

Que conforme a la información que obra en poder de esta Unidad Técnica, D. Jesús Pérez Sánchez-Toledo, con NIF 07989558W figura como *Investigador Principal* de los siguientes proyectos:

TITULO DEL PROYECTO: Utilidad clínica de la intervención temprana combinada con el método de medicina de precisión de 5 pasos (5SPM) en el primer episodio psicótico: El Proyecto CLUMP

REFERENCIA DEL PROYECTO: PI23/00582

FINANCIADOR: Instituto de Salud Carlos III

IMPORTE CONCEDIDO: 77.500,00 €

DURACIÓN: 01/01/2024 - 31/12/2026

Para que conste y a los efectos oportunos, se firma la presente en Salamanca, a 31 de enero de 2025

Instituto de Investigación Biomédica de Salamanca IBSAL Instituto de Investigación Biomédica de Salamanca 37007 Salamanca G37676360 Pº San Vicente 58-182 Edif. Virgen de la Vega

Fdo.: Luis García Ortiz

Director Científico del IBSAL

Complejo Asistencial Universitario de Salamanca. Edificio Virgen de la Vega, 10ª Planta. Pº de San Vicente 58-182, 37007 Salamanca.
Tel: 923291100 Ext. 55640/923090470/923090471 e-mail: proyectos@ibsal.es e-mail: admon.proyectos@ibsal.es
